# Evolution of ^18^F-FDG PET/CT Findings in Patients After COVID-19: An Initial Investigation

**DOI:** 10.2967/jnumed.121.262296

**Published:** 2022-02

**Authors:** Andrew Thornton, Francesco Fraioli, Simon Wan, Helen S. Garthwaite, Balaji Ganeshan, Robert I. Shortman, Raymond Endozo, Stefan Vöö, Irfan Kayani, Deena Neriman, Leon Menezes, Jamshed Bomanji, Toby Hilllman, Melissa Heightman, Joanna C. Porter, Ashley M. Groves

**Affiliations:** 1 Institute of Nuclear Medicine, UCLH/UCL, London, United Kingdom;; 2 ILD Service, UCLH/UCL Respiratory, London, United Kingdom; and; 3 Post-COVID Disease Service, UCLH, London, United Kingdom

**Keywords:** infectious disease, PET/CT, respiratory, ^18^F-FDG, COVID-19, PET/CT

## Abstract

The aim of this study was to assess the temporal evolution of pulmonary ^18^F-FDG uptake in patients with coronavirus disease 2019 (COVID-19) and post–COVID-19 lung disease (PCLD). **Methods:** Using our hospital’s clinical electronic records, we retrospectively identified 23 acute COVID-19, 18 PCLD, and 9 completely recovered ^18^F-FDG PET/CT patients during the 2 peaks of the U.K. pandemic. Pulmonary ^18^F-FDG uptake was measured as a lung target-to-background ratio (TBR_lung_ = SUV_max_/SUV_min_) and compared with temporal stage. **Results:** In acute COVID-19, less than 3 wk after infection, TBR_lung_ was strongly correlated with time after infection (*r*_s_ = 0.81, *P* < 0.001) and was significantly higher in the late stage than in the early stage (*P* = 0.001). In PCLD, TBR_lung_ was lower in patients treated with high-dose steroids (*P* = 0.003) and in asymptomatic patients (*P* < 0.001). **Conclusion:** Pulmonary ^18^F-FDG uptake in COVID-19 increases with time after infection. In PCLD, pulmonary ^18^F-FDG uptake rises despite viral clearance, suggesting ongoing inflammation. There was lower pulmonary ^18^F-FDG uptake in PCLD patients treated with steroids.

## Visual Abstract

**Figure:**
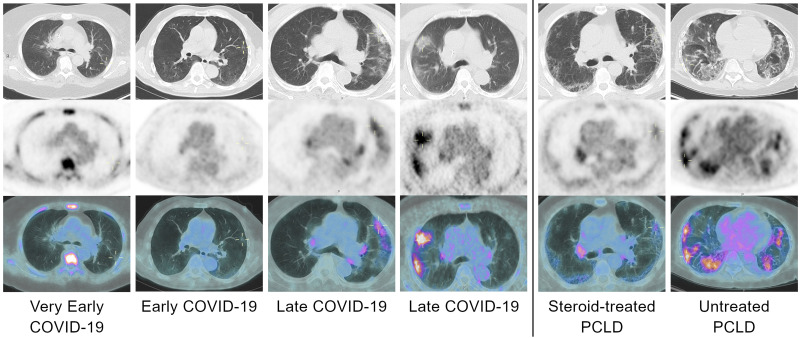

Throughout the United Kingdom, during February and March 2020 there was a rapid spread of coronavirus disease 2019 (COVID-19), which may result in viral pneumonitis and acute respiratory distress syndrome (*1*). The median time from symptom onset to intensive care admission was 10 d, although only 5% of patients were admitted (*1*). This is when antiviral responses are at a peak, suggesting that pneumonitis is a consequence of adaptive immunity (*2*).

Persistent respiratory symptoms affect at least one third of hospitalized COVID-19 patients, some of whom will have post–COVID-19 lung disease (PCLD) (*3*). Steroids are critical in reducing mortality from COVID-19, but their role in PCLD is less clear, and identifying those who might benefit may be difficult.

Currently, ^18^F-FDG PET/CT has no role in the management of patients with COVID-19 (*4*), and there has been little investigation into the quantification and evolution of ^18^F-FDG uptake in COVID-19 (Supplemental Table 1; supplemental materials are available at http://jnm.snmjournals.org). Given the growing role of ^18^F-FDG PET/CT in interstitial lung diseases, the primary aim of this preliminary study was to assess the temporal evolution of ^18^F-FDG uptake in COVID-19 and to correlate this evolution with clinical progression and recovery. A secondary aim was to investigate whether steroids could alter this evolution.

## MATERIALS AND METHODS

The Institutional Review Board approved this retrospective study and waived the requirement to obtain informed consent. The challenges of the pandemic constrained the methodologic design, necessitating a retrospective approach.

### Patient Selection

All studies performed in the department over the first U.K. peak of the coronavirus pandemic (March–April 2020) and from September 2020 to February 2021 (second peak) were assessed for acute COVID-19 by following the British Society of Thoracic Imaging guidelines or a confirmed history of COVID-19 in the electronic health record system (*5*). These studies included some of patients without positive polymerase chain reaction (PCR) test results, because of the poor availability of PCR tests in the early period. Also included were studies performed for persistent (>4 wk) respiratory symptoms, in keeping with PCLD, and studies of patients who had recovered from COVID-19 after the initial period. Ongoing treatment with steroids and other immunosuppressive drugs was recorded. Formal lung function tests were not performed because of infection risks. Acute studies between May and September 2020 were not examined because of the low prevalence and incidence of COVID-19 in London during that time (Supplemental Fig. 1; Supplemental Table 2).

### ^18^F-FDG PET/CT Imaging Protocol

Patients fasted for at least 6 h, and blood glucose levels were recorded before injection of 400 MBq of ^18^F-FDG adjusted for weight in keeping with the guidelines of the Administration of Radioactive Substances Advisory Committee (*6*). After an uptake time of 63.1 ± 10.9 min, whole-body PET scans of supine patients with their arms above their head were acquired at a rate of 2 min per bed position using a GE Healthcare Discovery 710 PET/CT scanner. A nonenhanced low-dose CT scan was acquired for anatomic coregistration and attenuation correction. Images were reconstructed using a resolution recovery iterative algorithm.

All images were reviewed by at least one dually accredited radiologist–nuclear medicine physician. Quantification was performed by investigators with at least 10 y of experience in quantifying PET/CT images of diffuse lung disease. PET analysis was performed with masking of clinical history and CT analysis.

### Determination of Temporal Stage

After review of the clinical, CT, and electronic health records, the number of days since disease onset was estimated, and the acute COVID-19 cases were assigned to 1 of 2 temporal groups: early or late COVID-19 (*7*). Early COVID-19 (approximately ≤1 wk after disease onset) was defined predominantly as CT findings of ground-glass opacities with or without associated interlobular thickening. Late COVID-19 (>1 wk to ≤4 wk after disease onset) was defined as CT findings of increasing consolidation and signs of resolution marked by subpleural sparing, development of a fibrous stripe, and crescentic consolidation or a reversed halo or atoll sign. Patients who were asymptomatic after 28 d were classed as recovered patients. In addition, patients who were imaged because of persistent symptoms after 28 d were described as having PCLD. The CT component was correlated with other cross-sectional images to reduce the likelihood of incorrect classification due to breathing artifacts.

### Quantitative ^18^F-FDG PET Analysis

All images were processed using a standard protocol on a dedicated imaging workstation (ADW Volume Viewer, version 4.6; GE Healthcare), which calculated the lung target-to-background ratio (TBR_lung_ = SUV_max_/SUV_mins_) following methods described previously (*8*–*10*).

### Statistics

The difference in ^18^F-FDG PET uptake measures within the lung against temporal staging and pretreatment with steroids were assessed using the nonparametric Mann–Whitney test. Results were depicted using box-and-whisker plots. All statistical analyses were performed using SPSS, version 25.0 (IBM).

## RESULTS

Of the 3,112 ^18^F-FDG PET/CT studies screened, 50 met the criteria for study entry, including 18 patients referred for ^18^F-FDG PET/CT for investigation of PCLD. Of these 50 patients (median age, 61 y; range, 18–87 y), 32 were male (64%), 27 were of ethnic minority background (54%), and 23 (46%) had acute COVID-19. None were intentionally imaged for COVID-19. Nine patients had asymptomatic recovered COVID-19 as confirmed by the electronic health record system (Supplemental Tables 3–5).

In 18 of the 50 patients, imaging was performed because of persistent shortness of breath and respiratory symptoms in keeping with PCLD. All 18 had been admitted to the hospital and had required oxygen. Fifteen of these patients previously had PCR tests positive for COVID-19, and COVID-19 was clinically diagnosed in the others. Nine had ongoing treatment with steroids for PCLD; the other 9 were not receiving treatment for their PCLD. All PCLD patients underwent repeated PCR testing confirming that they were PCR-negative before PET imaging (Supplemental Table 5).

### Temporal Stage

**FIGURE 1.**
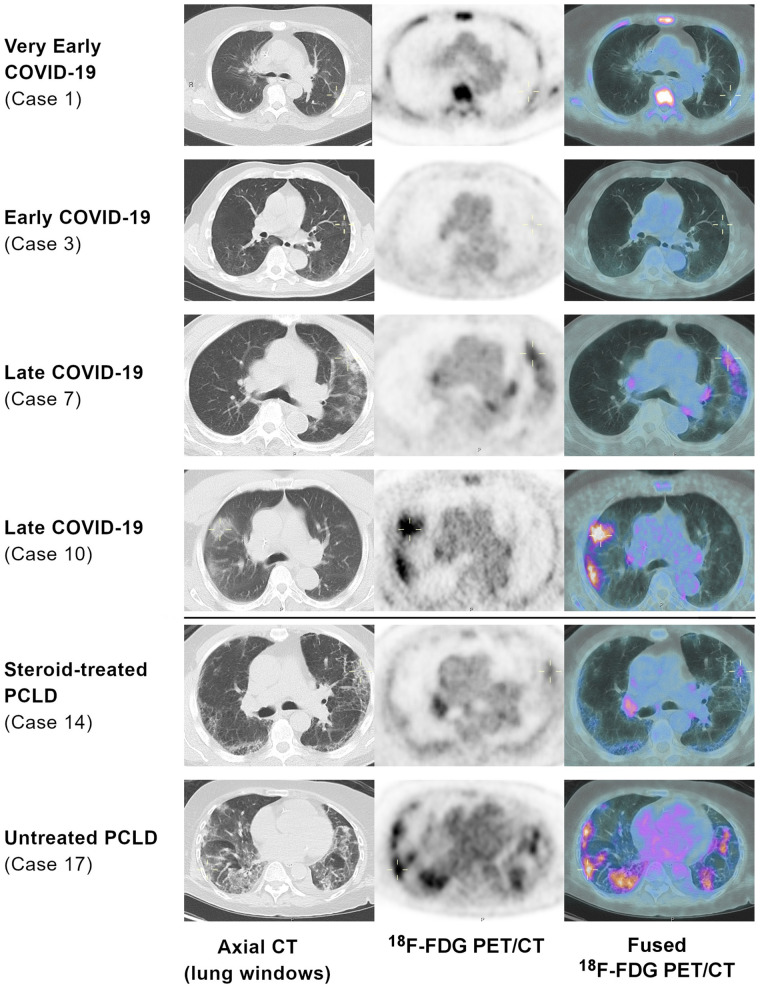
Exemplar images demonstrating increasing ^18^F-FDG uptake with temporal stage and lower ^18^F-FDG uptake in steroid-treated PCLD (lung-windowed axial CT, ^18^F-FDG PET [SUV 0–5], and ^18^F-FDG PET/CT images). Medullary uptake in case 1 was due to leukemia and not COVID-19.

After review of the CT component of the PET/CT (lung windows) and available clinical history, 8 (35%) of the 23 acute COVID-19 patients were determined to represent early COVID-19 and 15 (65%) late (Fig. 1; Supplemental Table 5).

### Association of Pulmonary ^18^F-FDG Uptake with Temporal Staging in Early- and Late-Stage Disease

**FIGURE 2.**
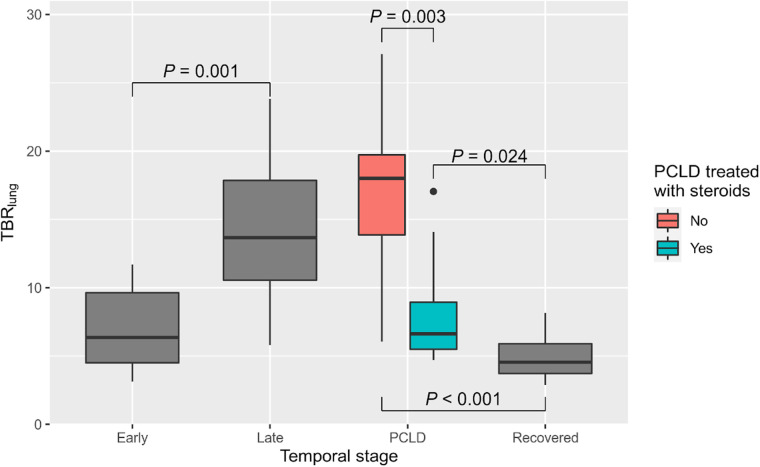
^18^F-FDG uptake (TBR_lung_) by temporal stage.

**FIGURE 3.**
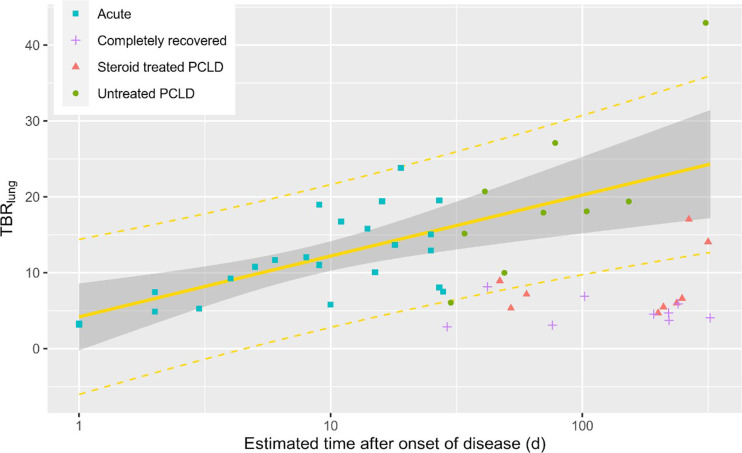
^18^F-FDG uptake (TBR_lung_) against estimated time after onset of disease (on logarithmic scale), with superimposed regression using 23 acute (early and late) patients (*F*_1,23_ = 14.94, *P* < 0.001; Spearman *r*_s_ = 0.595, *P* = 0.003). Steroid treated = at least 10 d of high-dose steroid treatment.

^18^F-FDG uptake analysis of lung lesions in acute-disease patients demonstrated an increasing TBR_lung_ over time, with progression from low-avidity ground-glass changes in the early stage to avid consolidation during the late stage (median values in the early stage: SUV_max_, 1.6, and TBR_lung_, 6.4; median values in the late stage: SUV_max_, 4.0, and TBR_lung_, 13.7). In acute-disease patients, TBR_lung_ differed significantly between the early and late stages, with late-stage patients having a higher TBR_lung_ than early-stage patients (*P* = 0.001; Fig. 2). Among these acute-disease patients, a significant positive correlation was observed between TBR_lung_ and estimated time since onset (*r*_s_ = 0.60, *P* = 0.003; Fig. 3). This correlation was stronger when limited to acute-disease patients estimated to be in the first 3 wk of infection (*n* = 18, *r*_s_ = 0.81, *P* < 0.001).

### Pulmonary ^18^F-FDG Uptake in PCLD

There was a lower TBR_lung_ in patients who had received treatment with high-dose steroids (*P* = 0.003) (Fig. 2) (median values in steroid-treated patients: SUV_max_, 2.4, and TBR_lung_, 6.62; median values in untreated patients: SUV_max_, 5.8, and TBR_lung_, 18.1).

TBR_lung_ was lower in asymptomatically recovered patients (median SUV_max_, 1.2; median TBR_lung_, 4.6) than in either untreated PCLD patients or those treated with steroids (*P* < 0.001 and *P* = 0.020, respectively; *P* < 0.001 on Kruskal–Wallis testing for all 3 groups).

## DISCUSSION

To our knowledge, this study was the first attempt to characterize the evolution of pulmonary ^18^F-FDG uptake in patients with COVID-19 assigned a temporal stage (early stage to late stage to PCLD) based on clinical context and CT findings.

The increase in lung avidity with time suggests increasing lung inflammation (*11*,*12*) in acute COVID-19. In most cases, ^18^F-FDG uptake would then be expected to decrease with viral clearance and establishment of immunity. There is, however, a subset of COVID-19 patients with delayed recovery who continue to show significant ^18^F-FDG uptake, reminiscent of our findings in interstitial lung disease (*8*,*9*,*13*,*14*), and raising the possibility that COVID-19 pneumonitis is associated with an activated host immune response rather than direct viral pathology (*12*,*15*,*16*). It would be useful to understand the ability of lung avidity to predict the clinical course or the likelihood that post–COVID-19 interstitial lung disease will develop in this patient cohort.

The RECOVERY study (Randomized Evaluation of COVID-19 Therapy), which this study predates, demonstrated a survival benefit from steroid use in hypoxic patients with COVID-19 (*15*). In our study, several patients went on to develop an inflammatory organizing pneumonia characterized by persistent and increasing ^18^F-FDG uptake. Steroid therapy is a recognized treatment for organizing pneumonia and other inflammatory interstitial lung diseases (*15*), and ^18^F-FDG uptake was consistently lower in those cases treated with postdischarge steroids. Our findings raise the question of whether steroid administration has a role not just in acute hypoxia but in the later stages of COVID-19 and in PCLD. This question has been debated (*15*), with calls for a randomized, controlled trial to define the role of steroid therapy more widely. Although imaging may be useful, it is hard to determine from CT whether parenchymal changes indicate reversible inflammation or irreversible fibrosis. It is possible that ^18^F-FDG PET/CT may offer a sensitive and specific biomarker to guide and rationalize steroid treatment.

Given the challenges of nuclear medicine imaging in the pandemic, this study has methodologic limitations. They are directly related to the infectious and emergent epidemic, the workload and severe capacity restraints of PET/CT departments, the need to protect staff and sterilize equipment, and the medical instability of seriously ill COVID-19 patients. These challenges limit patient numbers, preventing the use of a control group and longitudinal ^18^F-FDG PET/CT imaging. Diagnostic CT will likely remain the most practical way to investigate acute COVID-19, although PET imaging may give potential mechanistic insights. However, PCLD patients are not currently believed to be an infection risk, and performing longitudinal ^18^F-FDG PET/CT studies in this population may thus be realistic and feasible. This study was not prospectively designed to examine the use of steroids in PCLD; however, statistically significant lower ^18^F-FDG uptake was observed in PCLD patients who received steroids than in those who did not. Finally, the lack of PCR testing in the first wave, as well as the high incidence of asymptomatic cases throughout the pandemic, creates uncertainties about prevalence, and retrospective analyses may therefore suffer from selection bias. Despite the design limitations, the findings of this study offer some insight into the development of pulmonary disease in COVID-19 patients and can help provide the evidence to justify performing formal prospective studies on this topic in the future.

## CONCLUSION

^18^F-FDG uptake in COVID-19 patients increases with time after infection and correlates with severity. Persistent ^18^F-FDG uptake is seen in patients with PCLD disease. These findings suggest that future studies may be directed at the use of ^18^F-FDG PET/CT to clarify the disease trajectory and may aid management of those patients with persistent respiratory symptoms

## DISCLOSURE

Helen Garthwaite was funded by Breathing Matters. This work was undertaken at University College London Hospitals/University College London (UCLH/UCL), which receives funding from the U.K. Department of Health’s National Institute for Health Research Biomedical Research Centre’s funding scheme and the UCL Experimental Cancer Medicine Centre. No other potential conflict of interest relevant to this article was reported.

## Notes

KEY POINTS **QUESTION:** What is the temporal evolution of ^8^F-FDG uptake in COVID-19 and in PCLD? **PERTINENT FINDINGS:** ^18^F-FDG uptake was shown to increase with time after COVID-19 infection. Steroid treatment was associated with reduced uptake in PCLD. **IMPLICATIONS FOR PATIENT CARE:** ^18^F-FDG PET/CT may help us understand the disease trajectory and aid in management of PCLD.
